# The genome sequence of the Twin-spot Plume, *Stenoptilia bipunctidactyla* (Scopoli, 1763)

**DOI:** 10.12688/wellcomeopenres.19081.1

**Published:** 2023-02-24

**Authors:** William B.V. Langdon, University of Oxford and Wytham Woods Genome Acquisition Lab, Darwin Tree of Life Barcoding collective, Wellcome Sanger Institute Tree of Life programme, Wellcome Sanger Institute Scientific Operations: DNA Pipelines collective, Tree of Life Core Informatics collective, Darwin Tree of Life Consortium

**Affiliations:** 1 University of Oxford, Oxford, Oxfordshire, UK

**Keywords:** Stenoptilia bipunctidactyla, Twin-spot plume, genome sequence, chromosomal, Lepidoptera

## Abstract

We present a genome assembly from an individual male *Stenoptilia bipunctidactyla* (the Twin-spot Plume; Arthropoda; Insecta; Lepidoptera; Pterophoridae). The genome sequence is 822.9 megabases in span. Most of the assembly is scaffolded into 30 chromosomal pseudomolecules, including the Z sex chromosome. The mitochondrial genome has also been assembled and is 17.8 kilobases in length. Gene annotation of this assembly on Ensembl has identified 22,137 protein coding genes.

## Species taxonomy

Eukaryota; Metazoa; Ecdysozoa; Arthropoda; Hexapoda; Insecta; Pterygota; Neoptera; Endopterygota; Lepidoptera; Glossata; Ditrysia; Pterophoroidea; Pterophoridae; Pterophorinae; *Stenoptilia*; *Stenoptilia bipunctidactyla* ( Scopoli, 1763) (NCBI:txid1660692).

## Background

*Stenoptilia bipunctidactyla* ( Scopoli, 1763), known as the ‘Twin-Spot Plume’ ( Hart, 2011), is a micro moth of the family Pterophoridae, commonly known as ‘Plume moths’ for their thin, elongated wings which are often held rolled-up when at rest, at right angles to the body.

It is a relatively widespread member of the group, known from all mainland European countries ( Gielis, no date), but precise understanding of its range and biology have been clouded by the relatively recent description of a number of other species formerly included under *S. bipunctidactyla* ( Arenberger, 2005; Gielis, 1996; Gielis, 2003). Adults of this ‘ *S. bipunctidactyla* complex’ are extremely similar externally, but species can be separated on the basis of their genital morphology, and to some extent their foodplants. Five of the species described in this complex occur in Europe alongside the original *S. bipunctidactyla* ( Rennwald, no date), although *S. succisae* ( Gibeaux & Nel, 1991) has recently been reduced back to a synonym of *S. bipunctidactyla* by ( Huemer *et al.*, 2021). In the UK, three members of the complex are known, of which *S. bipunctidactyla* appears to be by far the most widespread. *S. annadactyla* (‘Small Scabious Plume’) is currently known only from the Brecklands of East Anglia, while *S. scabiodactyla* (‘Gregson’s Plume’) appears to be a more northern species, but its distribution is poorly known ( Emmet *et al.*, no date).

Confusion over the taxonomy of *S. bipunctidactyla* has also fuelled confusion over its biology ( Rennwald, no date), but in the UK it has two main foodplants – Field Scabious ( *Knautia arvensis* L.) and Devil’s Bit Scabious ( *Succisa pratensis* Moench) ( Hart, 2011). In the south of England there are two broods a year, with larvae of the first brood emerging from hibernation (probably at the base of the plant) in early spring ( Hart, 2011). On *K. arvensis* they then mine a growing stem, causing blackening near the tips of the newest leaves, while on *S .pratensis* larvae initially mine the midrib of an old leaf, before feeding under the new leaves, spinning them together as they grow older ( Hart, 2011). Adults then fly in late May and June, and second brood larvae can be found from late June to mid-August, feeding concealed inside the flowers of the foodplants, with another brood of adults in late July and August ( Hart, 2011).

In the UK, both foodplants of *S. bipunctidactyla* are species of un-improved, low-nutrient grasslands, and though the moth is widespread it has probably declined as a result of the improvement of such habitats ( Hart, 2011). *K. arvensis* occurs mostly on drier, often calcareous sites, and *S. pratensis* in similar habitats, but also on damper neutral and acid grasslands. *S. bipunctidactyla* occurs in all these habitats.

The genome of *Stenoptilia bipunctidactyla* was sequenced as part of the Darwin Tree of Life Project, a collaborative effort to sequence all named eukaryotic species in the Atlantic Archipelago of Britain and Ireland. Here we present a chromosomally complete genome sequence for *Stenoptilia bipunctidactlya*, based on one male specimen from Wytham Woods, Oxfordshire, UK. This specimen was captured in the area of the woods known as ‘Upper Seeds’ where *S. bipunctidactyla* larvae have been found feeding on Field Scabious.

## Genome sequence report

**Figure 1.**
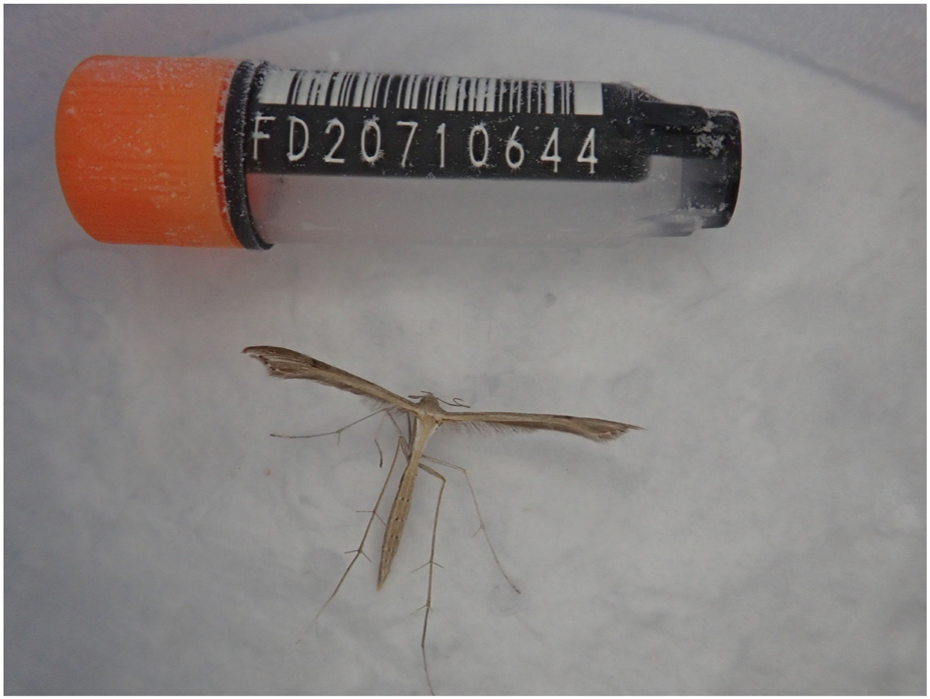
Photograph of the *Stenoptilia bipunctidactyla* (ilSteBipu1) specimen used for genome sequencing.

The genome was sequenced from one male *S. bipunctidactyla* ( Figure 1) collected from Wytham Woods, UK (latitude 51.77, longitude –1.33). A total of 24-fold coverage in Pacific Biosciences single-molecule HiFi long reads was generated. Primary assembly contigs were scaffolded with chromosome conformation Hi-C data. Manual assembly curation corrected 76 missing or mis-joins and removed 41 haplotypic duplications, reducing the assembly length by 2.8% and the scaffold number by 7.4%, reducing the scaffold N50 by 0.51%.

**Figure 2.**
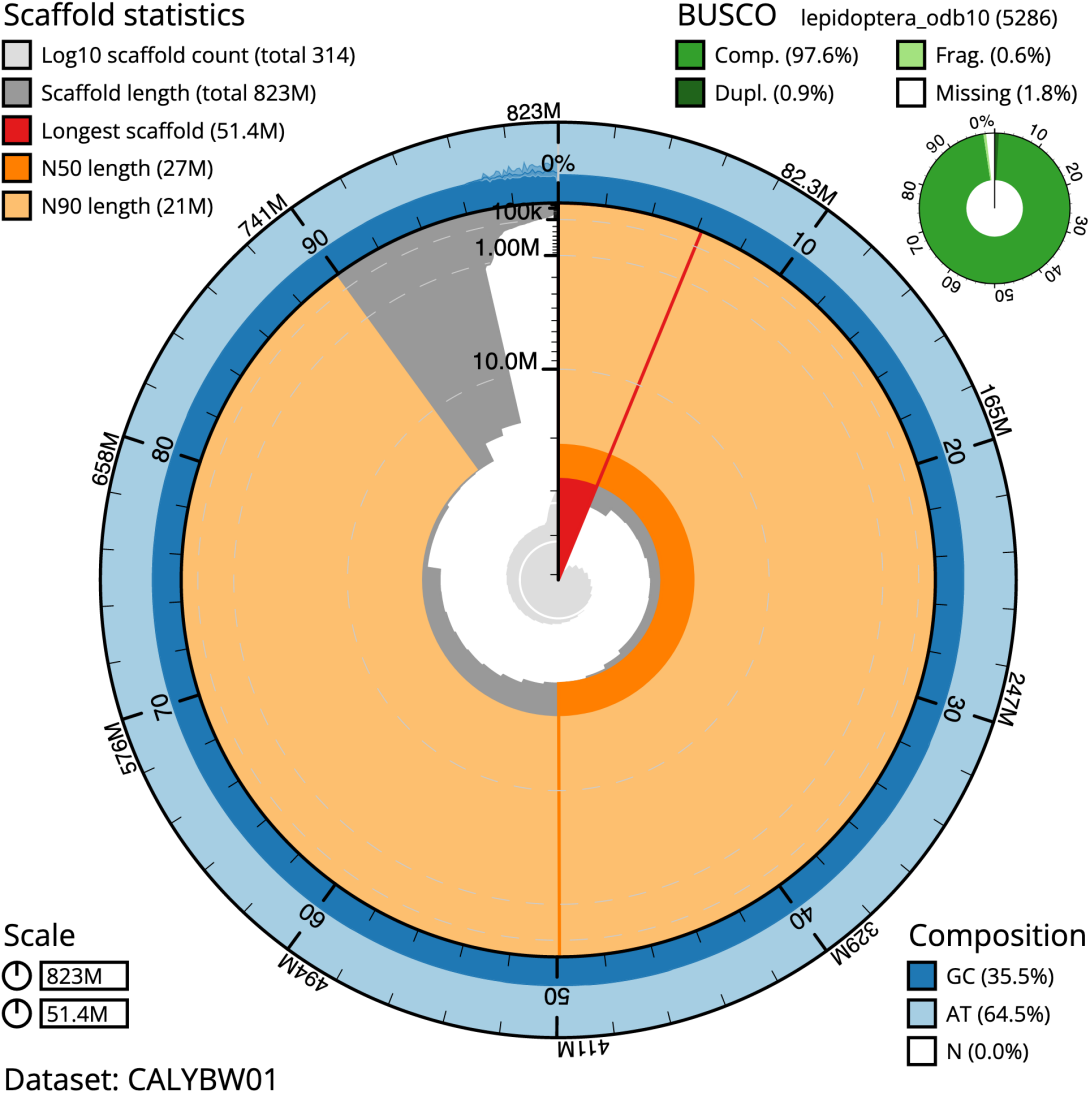
Genome assembly of *Stenoptilia bipunctidactyla*, ilSteBipu1.1: metrics. The BlobToolKit Snailplot shows N50 metrics and BUSCO gene completeness. The main plot is divided into 1,000 size-ordered bins around the circumference with each bin representing 0.1% of the 822,897,450 bp assembly. The distribution of scaffold lengths is shown in dark grey with the plot radius scaled to the longest scaffold present in the assembly (51,424,521 bp, shown in red). Orange and pale-orange arcs show the N50 and N90 scaffold lengths (27,025,175 and 20,980,724 bp), respectively. The pale grey spiral shows the cumulative scaffold count on a log scale with white scale lines showing successive orders of magnitude. The blue and pale-blue area around the outside of the plot shows the distribution of GC, AT and N percentages in the same bins as the inner plot. A summary of complete, fragmented, duplicated and missing BUSCO genes in the lepidoptera_odb10 set is shown in the top right. An interactive version of this figure is available at https://blobtoolkit.genomehubs.org/view/ilSteBipu1.1/dataset/CALYBW01/cumulative.

The final assembly has a total length of 822.9 Mb in 313 sequence scaffolds with a scaffold N50 of 27.0 Mb ( Table 1). Most (96.41%) of the assembly sequence was assigned to 30 chromosomal-level scaffolds, representing 29 autosomes and the Z sex chromosome. Chromosome-scale scaffolds confirmed by the Hi-C data are named in order of size. ( Figure 2– Figure 5; Table 2). The assembly has a BUSCO v5.3.2 ( Manni *et al.*, 2021) completeness of 97.6%% (single 96.7%, duplicated 0.9%) using the lepidoptera_odb10 reference set. While not fully phased, the assembly deposited is of one haplotype. Contigs corresponding to the second haplotype have also been deposited.

**Table 1.** Genome data for *Stenoptilia bipunctidactyla*, ilSteBipu1.1.

Project accession data		
Assembly identifier	ilSteBipu1.1	
Species	*Stenoptilia bipunctidactyla*	
Specimen	ilSteBipu1	
NCBI taxonomy ID	1660692	
BioProject	PRJEB53159	
BioSample ID	SAMEA10979057	
Isolate information	ilSteBipu1 (PacBio) ilSteBipu2 (Hi-C) ilSteBipu3 (RNA-Seq)	
Assembly metrics *		*Benchmark*
Consensus quality (QV)	65.4	*≥ 50*
*k*-mer completeness	100%	*≥ 95%*
BUSCO **	C:97.6%[S:96.7%,D:0.9%], F:0.6%,M:1.8%,n:5,286	*C ≥ 95%*
Percentage of assembly mapped to chromosomes	96.41%	*≥ 95%*
Sex chromosomes	Z chromosome	*localised homologous pairs*
Organelles	Mitochondrial genome assembled	*complete single alleles*
Raw data accessions		
PacificBiosciences SEQUEL II	ERR9836416	
Hi-C Illumina	ERR9793170	
PolyA RNA-Seq Illumina	ERR10123705	
Genome assembly		
Assembly accession	GCA_944452665.1	
*Accession of alternate haplotype*	GCA_944452705.1	
Span (Mb)	822.9	
Number of contigs	675	
Contig N50 length (Mb)	3.7	
Number of scaffolds	313	
Scaffold N50 length (Mb)	27.0	
Longest scaffold (Mb)	51.4	
**Genome annotation**		
Number of protein-coding genes	22,137	
Number of gene transcripts	22,313	

* Assembly metric benchmarks are adapted from column VGP-2020 of “Table 1: Proposed standards and metrics for defining genome assembly quality” from ( Rhie *et al.*, 2021). ** BUSCO scores based on the lepidoptera_odb10 BUSCO set using v5.3.2. C = complete [S = single copy, D = duplicated], F = fragmented, M = missing, n = number of orthologues in comparison. A full set of BUSCO scores is available at https://blobtoolkit.genomehubs.org/view/ilSteBipu1.1/dataset/CALYBW01/busco.

**Figure 3.**
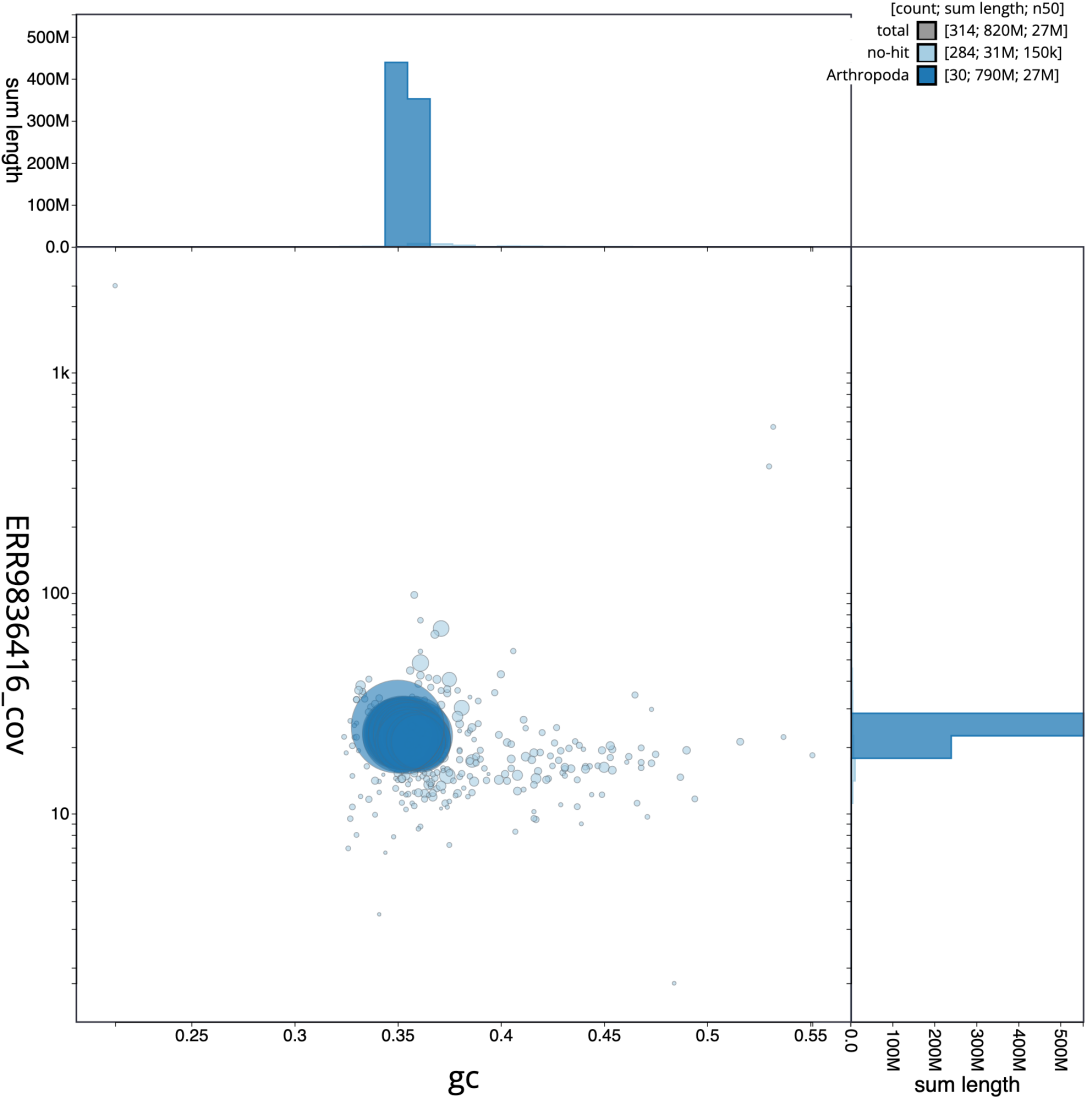
Genome assembly of *Stenoptilia bipunctidactyla*, ilSteBipu1.1: GC coverage. BlobToolKit GC-coverage plot. Scaffolds are coloured by phylum. Circles are sized in proportion to scaffold length. Histograms show the distribution of scaffold length sum along each axis. An interactive version of this figure is available at https://blobtoolkit.genomehubs.org/view/ilSteBipu1.1/dataset/CALYBW01/blob.

**Figure 4.**
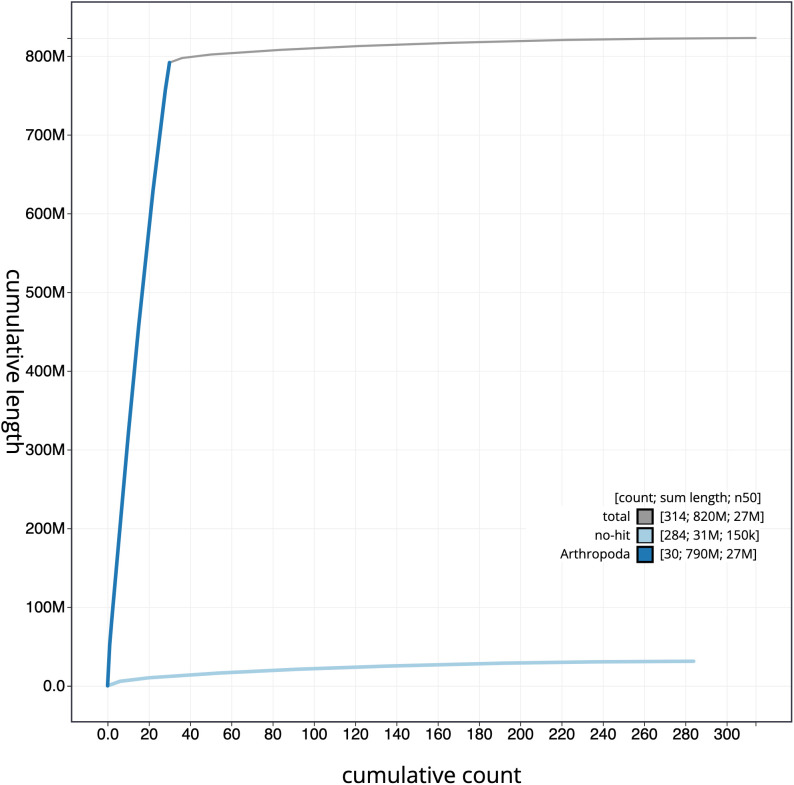
Genome assembly of *Stenoptilia bipunctidactyla*, ilSteBipu1.1: cumulative sequence. BlobToolKit cumulative sequence plot. The grey line shows cumulative length for all scaffolds. Coloured lines show cumulative lengths of scaffolds assigned to each phylum using the buscogenes taxrule. An interactive version of this figure is available at https://blobtoolkit.genomehubs.org/view/ilSteBipu1.1/dataset/CALYBW01/cumulative.

**Figure 5.**
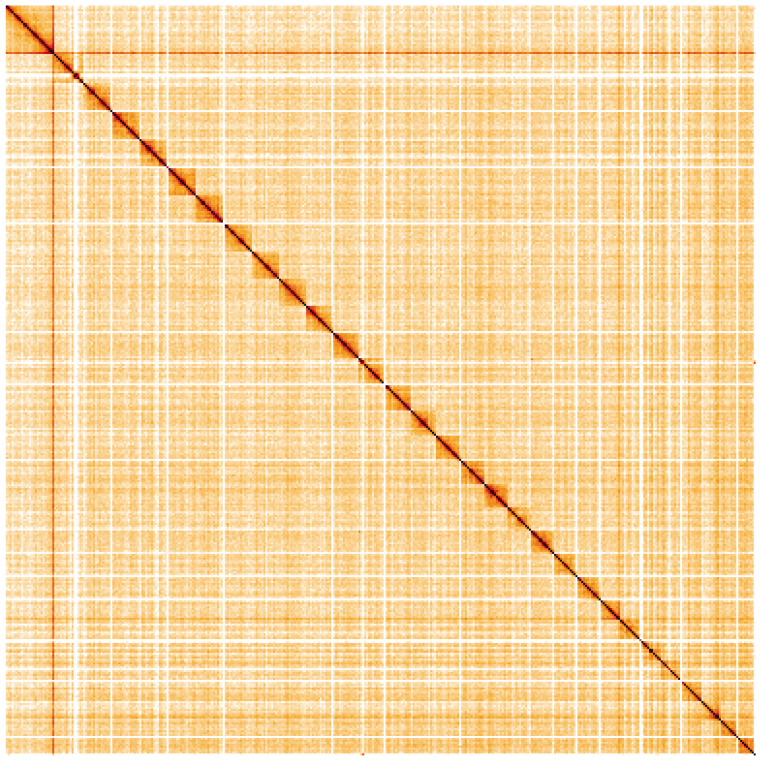
Genome assembly of *Stenoptilia bipunctidactyla*, ilSteBipu1.1: Hi-C contact map. Hi-C contact map of the ilSteBipu1.1 assembly, visualised using HiGlass. Chromosomes are shown in order of size from left to right and top to bottom. An interactive version of this figure may be viewed at https://genome-note-higlass.tol.sanger.ac.uk/l/?d=Dv3cV6lwQC-nrfcURO3Aew.

**Table 2.** Chromosomal pseudomolecules in the genome assembly of *Stenoptilia bipunctidactyla*, ilSteBipu1.

INSDC accession	Chromosome	Size (Mb)	GC%
OX101722.1	1	31.85	35.1
OX101723.1	2	30.1	35.3
OX101724.1	3	29.91	35.2
OX101725.1	4	29.8	35.5
OX101726.1	5	29.39	35.2
OX101727.1	6	29.22	35
OX101728.1	7	29.08	35.1
OX101729.1	8	29.06	35.8
OX101730.1	9	29.04	35.3
OX101731.1	10	28.3	35.3
OX101732.1	11	27.55	35.4
OX101733.1	12	27.16	36
OX101734.1	13	27.03	35.6
OX101735.1	14	26.6	35.2
OX101736.1	15	25.82	35.2
OX101737.1	16	25.22	35.5
OX101738.1	17	24.88	35.4
OX101739.1	18	24.68	35.6
OX101740.1	19	24.43	35.4
OX101741.1	20	24.19	35.6
OX101742.1	21	24.13	35.6
OX101743.1	22	21.66	35.9
OX101744.1	23	21.69	35.5
OX101745.1	24	21.69	35.4
OX101746.1	25	21.23	36.1
OX101747.1	26	21.12	36.1
OX101748.1	27	20.98	35.5
OX101749.1	28	17.77	35.8
OX101750.1	29	16.68	36
OX101721.1	Z	51.42	35
OX101751.1	MT	0.02	21.3
-	unplaced	31.2	38.2

## Genome annotation report

The *S. bipunctidactyla* genome assembly (GCA_944452665.1) was annotated using the Ensembl rapid annotation pipeline ( Table 1; https://rapid.ensembl.org/Stenoptilia_bipunctidactyla_GCA_944452665.1/). The resulting annotation includes 22,313 transcribed mRNAs from 22,137 protein-coding genes.

## Methods

### Sample acquisition and nucleic acid extraction

Three *Stenoptilia bipunctidactyla* specimens (ilSteBipu1, ilSteBipu2 and ilSteBipu3) were collected in Wytham Woods, Oxfordshire (biological vice-county: Berkshire), UK (latitude 51.77, longitude –1.33) on 10 August 2021. The specimens were taken from a grassland habitat by netting and were snap-frozen on dry ice. William Langdon (University of Oxford) collected and identified the specimens.

DNA was extracted at the Tree of Life laboratory, Wellcome Sanger Institute (WSI). The ilSteBipu1 sample was weighed and dissected on dry ice with tissue set aside for Hi-C sequencing. Whole organism tissue was disrupted using a Nippi Powermasher fitted with a BioMasher pestle. High molecular weight (HMW) DNA was extracted using the Qiagen MagAttract HMW DNA extraction kit. HMW DNA was sheared into an average fragment size of 12–20 kb in a Megaruptor 3 system with speed setting 30. Sheared DNA was purified by solid-phase reversible immobilisation using AMPure PB beads with a 1.8X ratio of beads to sample to remove the shorter fragments and concentrate the DNA sample. The concentration of the sheared and purified DNA was assessed using a Nanodrop spectrophotometer and Qubit Fluorometer and Qubit dsDNA High Sensitivity Assay kit. Fragment size distribution was evaluated by running the sample on the FemtoPulse system.

RNA was extracted from ilSteBipu3 in the Tree of Life Laboratory at the WSI using TRIzol, according to the manufacturer’s instructions. RNA was then eluted in 50 μl RNAse-free water and its concentration was assessed using a Nanodrop spectrophotometer and Qubit Fluorometer using the Qubit RNA Broad-Range (BR) Assay kit. Analysis of the integrity of the RNA was done using Agilent RNA 6000 Pico Kit and Eukaryotic Total RNA assay.

### Sequencing

Pacific Biosciences HiFi circular consensus DNA sequencing libraries were constructed according to the manufacturers’ instructions. Poly(A) RNA-Seq libraries were constructed using the NEB Ultra II RNA Library Prep kit. DNA and RNA sequencing were performed by the Scientific Operations core at the WSI on Pacific Biosciences SEQUEL II (HiFi) and Illumina NovaSeq 6000 (RNA-Seq) instruments. Hi-C data were also generated from ilSteBipu2 using the Arima v2 kit and sequenced on the Illumina NovaSeq 6000 instrument.

### Genome assembly

Assembly was carried out with Hifiasm ( Cheng *et al.*, 2021) and haplotypic duplication was identified and removed with purge_dups ( Guan *et al.*, 2020). The assembly was then scaffolded with Hi-C data ( Rao *et al.*, 2014) using YaHS ( Zhou *et al.*, 2023). The assembly was checked for contamination and corrected as described previously ( Howe *et al.*, 2021). Manual curation was performed using HiGlass ( Kerpedjiev *et al.*, 2018) and Pretext ( Harry, 2022). The mitochondrial genome was assembled using MitoHiFi ( Uliano-Silva *et al.*, 2022), which performed annotation using MitoFinder ( Allio *et al.*, 2020). The genome was analysed and BUSCO scores were generated within the BlobToolKit environment ( Challis *et al.*, 2020). Table 3 contains a list of all software tool versions used, where appropriate.

**Table 3.** Software tools and versions used.

Software tool	Version	Source
BlobToolKit	3.5.2	Challis *et al.*, 2020
Hifiasm	0.16.1-r375	Cheng *et al.*, 2021
HiGlass	1.11.6	Kerpedjiev *et al.*, 2018
MitoHiFi	2	Uliano-Silva *et al.*, 2022
PretextView	0.2	Harry, 2022
purge_dups	1.2.3	Guan *et al.*, 2020
YaHS	yahs-1.1.91eebc2	Zhou *et al.*, 2023

### Genome annotation

The BRAKER2 pipeline ( Brůna *et al.*, 2021) was used in the default protein mode to generate annotation for the *S. bipunctidactyla* assembly (GCA_944452665.1) in Ensembl Rapid Release.

### Ethics and compliance issues

The materials that have contributed to this genome note have been supplied by a Darwin Tree of Life Partner. The submission of materials by a Darwin Tree of Life Partner is subject to the Darwin Tree of Life Project Sampling Code of Practice. By agreeing with and signing up to the Sampling Code of Practice, the Darwin Tree of Life Partner agrees they will meet the legal and ethical requirements and standards set out within this document in respect of all samples acquired for, and supplied to, the Darwin Tree of Life Project. All efforts are undertaken to minimise the suffering of animals used for sequencing. Each transfer of samples is further undertaken according to a Research Collaboration Agreement or Material Transfer Agreement entered into by the Darwin Tree of Life Partner, Genome Research Limited (operating as the Wellcome Sanger Institute), and in some circumstances other Darwin Tree of Life collaborators.

## Data Availability

European Nucleotide Archive: *Stenoptilia bipunctidactyla* (twin-spot plume). Accession number PRJEB53159; https://identifiers.org/ena.embl/PRJEB53159. ( Wellcome Sanger Institute, 2022) The genome sequence is released openly for reuse. The *Stenoptilia bipunctidactyla* genome sequencing initiative is part of the Darwin Tree of Life (DToL) project. All raw sequence data and the assembly have been deposited in INSDC databases. Raw data and assembly accession identifiers are reported in Table 1.

## Author information

Members of the University of Oxford and Wytham Woods Genome Acquisition Lab are listed here: https://doi.org/10.5281/zenodo.4789928.

Members of the Darwin Tree of Life Barcoding collective are listed here: https://doi.org/10.5281/zenodo.4893703.

Members of the Wellcome Sanger Institute Tree of Life programme are listed here: https://doi.org/10.5281/zenodo.4783585.

Members of Wellcome Sanger Institute Scientific Operations: DNA Pipelines collective are listed here: https://doi.org/10.5281/zenodo.4790455.

Members of the Tree of Life Core Informatics collective are listed here: https://doi.org/10.5281/zenodo.5013541.

Members of the Darwin Tree of Life Consortium are listed here: https://doi.org/10.5281/zenodo.4783558.
