# Supplementary material for: The genome sequence of the Twin-spot Plume, Stenoptilia bipunctidactyla (Scopoli, 1763)
Source: Wellcome Open Res. 2023 Feb 24;8:103. [Version 1] doi: 10.12688/wellcomeopenres.19081.1 (PMC10548107; doi:10.12688/wellcomeopenres.19081.1)
Supplement: Peer review history

# Reviewer report: Reviewer response for version 1

**DOI:** 10.21956/wellcomeopenres.21156.r64390

**Published:** 2023-10-03

**Authors:** Wendy A Valencia-Montoya

**Affiliations:** 1 Department of Organismic and Evolutionary Biology and Museum of Comparative Zoology, Harvard University, Cambridge, Massachusetts, USA

The researchers present a chromosome-level genome assembly of the “Twin-Spot Plume” moth, *Stenoptilia bipunctidactyla*. The authors employed state-of-the-art methods for sequencing, assembling, and annotation of insect genomes. The paper is clearly written, including information about other moth species in the same complex along with observations on their natural history. There are just some minor comments:

1. Some references are currently missing the date. For instance: (Gielis, no date) and (Rennwald, no date).
2. This sentence might not be clear to the general public: “In the UK, both foodplants of *S. bipunctidactyla* are species of un-improved, low-nutrient grasslands, and though the moth is widespread it has probably declined as a result of the improvement of such habitats (Hart, 2011).” Maybe explaining what “un-improved” and “improvement” refer to in this context would help.
3. It would be useful to cite the link for the lepidoptera_odb10 database.
4. Do the authors also use transcriptomic data for annotation as part of their annotation pipeline? If this is the case, it would be important to include this information.

Are sufficient details of methods and materials provided to allow replication by others?

Yes

Is the rationale for creating the dataset(s) clearly described?

Yes

Are the datasets clearly presented in a useable and accessible format?

Yes

Are the protocols appropriate and is the work technically sound?

Yes

Reviewer Expertise:

Evolutionary Biology

I confirm that I have read this submission and believe that I have an appropriate level of expertise to confirm that it is of an acceptable scientific standard.

# Reviewer report: Reviewer response for version 1

**DOI:** 10.21956/wellcomeopenres.21156.r55892

**Published:** 2023-06-05

**Authors:** Luciana Bolsoni Lourenço

**Affiliations:** 1 Laboratório de Estudos Cromossômicos (LabEsC), Departamento de Biologia Estrutural e Funcional, Instituto de Biologia, Universidade Estadual de Campinas, Campinas, State of São Paulo, Brazil

The text is clear and the data are very useful. The figures are informative and the datasets can be easily accessed. I would only suggest that the authors indicate how they identified the sex chromosome. Is there any previous genetic or cytogenetic paper that might support the identification of the Z chromosome?

Are sufficient details of methods and materials provided to allow replication by others?

Yes

Is the rationale for creating the dataset(s) clearly described?

Yes

Are the datasets clearly presented in a useable and accessible format?

Yes

Are the protocols appropriate and is the work technically sound?

Yes

Reviewer Expertise:

Cytogenomics

I confirm that I have read this submission and believe that I have an appropriate level of expertise to confirm that it is of an acceptable scientific standard.
